# Testing EGFR with Idylla on Cytological Specimens of Lung Cancer: A Review

**DOI:** 10.3390/ijms22094852

**Published:** 2021-05-03

**Authors:** Alessandro Caputo, Angela D’Ardia, Francesco Sabbatino, Caterina Picariello, Chiara Ciaparrone, Pio Zeppa, Antonio D’Antonio

**Affiliations:** 1 Department of Medicine and Surgery, University of Salerno, 84081 Salerno, Italy; adardia@unisa.it (A.D.); fsabbatino@unisa.it (F.S.); pzeppa@unisa.it (P.Z.); 2 Department of Pathology, University Hospital “San Giovanni di Dio e Ruggi D’Aragona”, 84125 Salerno, Italy; caterinapicariello@gmail.com (C.P.); chiaraciaparrone2@gmail.com (C.C.); andantonio@unisa.it (A.D.)

**Keywords:** idylla, cytology, cytopathology, epidermal growth factor receptor, lung cancer, lung adenocarcinoma

## Abstract

The current standard of care for advanced non-small-cell lung cancer is based on detecting actionable mutations that can benefit from targeted therapy. Comprehensive genetic tests can have long turn-around times, and because EGFR mutations are the most prevalent actionable mutation, a quick detection would enable a prompt initiation of targeted therapy. Furthermore, the scarcity of diagnostic material means that sometimes only cytologic material is available. The Idylla™ EGFR assay is a real-time PCR–based method able to detect 51 EGFR mutations in 2.5 h. Idylla is validated for use only on FFPE sections, but some researchers described their experiences with cytological material. We reviewed the relevant literature, finding four articles describing 471 cases and many types of cytological input material: smears, cell-block sections, suspensions, and extracted DNA. The sensitivity, specificity, and limit of detection appear comparable to those obtained with histological input material, with one exception: the usage of scraped stained smears as input may reduce the accuracy of the test. In conclusion, usage of cytological material as input to the Idylla EGFR test is possible. A workflow where common mutations are tested first and fast, leaving rarer mutations for subsequent comprehensive profiling, seems the most effective approach.

## 1. Introduction

Lung cancer is the leading cause of cancer death, accounting for 40% of all cancer-related deaths [1,2]. The two most common histological subtypes of NSCLC are adenocarcinoma (60%) and squamous cell carcinoma (15%), with mixed and large cell tumors representing uncommon variants [3,4]. Despite advances in early diagnosis and screening, most cases are currently diagnosed at an advanced stage, where the disease is metastatic and surgical curative treatment is no longer feasible [5]. Approximately one-third of NSCLCs, especially non-squamous NSCLCs, harbor an actionable mutation that can be treated with target therapy [6,7,8].

Thanks to this, the treatment options for advanced non-small-cell lung cancer (NSCLC) are improving: in the early 2000s, advanced NSCLC had a 5-year overall survival of about 5%; now, some groups of patients reach 5-year overall survivals as high as 40% [6,9,10]. Targeted therapies are effective only in some subgroups of patients, so pathologists and molecular biologists are increasingly called to select these patients [11]. Additionally, surgical resection is not recommended for advanced NSCLC, so the molecular assessment must be performed on ever-shrinking small biopsies or, often, on cytological material [12,13,14,15,16]. Recent guidelines recommend molecular phenotyping of advanced NSCLCs to guide therapy [17,18,19]. The identification of a targetable mutation implies the possibility to treat the patient with a more effective therapy than conventional cytotoxic chemotherapy [20,21]. As our knowledge of rarer mutations increases and new drugs become available, guidelines suggest testing an increasing number of them [17,20,22,23,24]. Most current guidelines recommend testing for at least epidermal growth factor receptor (EGFR) mutations, v-Raf murine sarcoma viral oncogene homolog B (BRAF) V600E mutation, neurotrophic receptor tyrosine kinase (NTRK) gene fusions, and anaplastic lymphoma kinase (ALK) and ROS1 rearrangements, in addition to immunohistochemical evaluation of the programmed death-ligand 1 (PD-L1) tumor proportion score (TPS) [6,11,12,13,17,20,25,26]. The testing of numerous other alterations is recommended or suggested by some guidelines, as they are either already targetable or in the process of becoming targetable (e.g., a phase II/III trial for targeted therapy is in progress) [12,27,28]. These include human epidermal growth factor receptor 2 (HER2) amplification and mutations, MET amplification, RET rearrangements, fibroblast growth factor receptor (FGFR) amplifications, and translocations, and Kirsten rat sarcoma virus (KRAS) G12C point mutations [12,29,30,31,32,33].

EGFR mutations are the most common targetable mutations in NSCLC (15–40%) [34,35,36,37]. The EGFR tyrosine kinase receptor is the first member of the HER (human epidermal growth factor receptor) or ErbB (avian erythroblastosis oncogene B) family, which consists of four members and includes HER2/ErbB2 [38,39,40]. HER receptors regulate pathways involved in cell proliferation and apoptosis, as well as cell motility and neovascularization [41,42]. Activation depends on receptor dimerization and leads to the activation of two main downstream signaling pathways: PIK3CA/AKT1/mTOR and RAS/RAF/MAPK [43,44,45,46]. These pathways are crucial in oncogenesis, and in fact, they are commonly mutated in human cancers [47,48].

EGFR mutations are more commonly found in NSCLCs with adenocarcinoma histology, in women and in never smokers [49,50,51]. Most mutations affect exons 18–21 and are represented by exon 19 deletions and L858R (exon 21) point mutations (together accounting for 90% of EGFR mutations) [52]. Tumors harboring these mutations can be treated with EGFR tyrosine kinase inhibitors (EGFR-TKIs) such as gefitinib and erlotinib (first generation), afatinib and dacomitinib (second generation), and osimertinib (third generation), obtaining significantly longer progression-free survivals compared with conventional cytotoxic chemotherapy [53,54,55,56,57,58]. However, treatment with first- and second-generation EGFR-TKIs tends to select an additional mutation (EGFR T790M) which is associated with treatment resistance [59,60]. Osimertinib (a third-generation EGFR-TKI) is at present the only drug that can overcome this resistance [61,62]. It is thus currently indicated for the treatment of EGFR T790M-mutated NSCLC and has shown promising data even as a first-line EGFR-TKI [63]. However, EGFR mutations that confer resistance to osimertinib are starting to emerge [64].

Due to long turn-around times for comprehensive molecular phenotyping (sometimes made even longer by the need to outsource), many patients are treated with conventional chemotherapy despite potentially harboring an actionable mutation. Some cases may be detected and treated earlier by performing an initial fast and specific test focused on the most commonly involved gene—EGFR [7].

The Idylla™ EGFR assay is one such test. It is a cartridge-based closed system that assesses 51 mutations using real-time PCR, with a turn-around time of 2.5 h and a hands-on time of 2 min. The system takes care of deparaffinization and nucleic acid extraction, so slices cut from the FFPE block can be directly inserted into the cartridge. However, samples other than FFPE slices can be employed as inputs, and some researchers have reported their experience with cytological material such as scraped smears and suspensions of fine-needle biopsy material. This review aims to gather, contextualize and summarize the evidence about the usage of the Idylla™ EGFR assay in cytological specimens of NSCLC.

## 2. Results

In total, 220 articles were identified during the initial search. After duplicate removal and title screening, 14 articles were left. After abstract and full-text screening, 3 articles were left [7,65,66]. Hand-searching of references did not yield additional articles. Unpublished data from a submitted manuscript by our group was included (D’Ardia et al. 2021). Ultimately, 4 articles describing 471 cases were included in the present review [7,65,66].

## 3. Discussion

To assess the adequacy and efficacy of the Idylla™ system in testing EGFR mutations in cytological specimens of non-small-cell lung cancer (NSCLC), we reviewed the international literature identifying 4 articles describing 471 cases [7,65,66]. Overall, Idylla appears to be adequate and effective on the whole range of cytological samples (smears, cell blocks, residual material), with a few important pitfalls that will be discussed.

### 3.1. Diagnostic Performance of Idylla on Cytological Material

Arcila and colleagues [7] formally investigated the sensitivity and specificity of Idylla on cytological material compared with next-generation sequencing (NGS). In a validation phase, they found a sensitivity of 94.0% (95% CI: 83.5–98.8%) and a specificity of 100% (89.4–100.0%). In a subsequent clinical implementation phase, they report a sensitivity of 95.6% (84.9–99.5%) and a specificity of 100%. The other studies either did not assess sensitivity and specificity or did so but with an older reference method [65,67]. These results are comparable to those obtained with histological samples [68].

Regarding the analytical sensitivity (limit of detection—LOD), Arcila and colleagues report that it depends on the total input DNA, on the variant allele frequency (VAF), and on the specific variant tested. Most mutations can be detected at a 1.5–2.2% VAF with 50 ng input DNA; VAFs as low as 0.4% can be detected with higher DNA inputs (400 ng) [7]. De Luca and colleagues report a LOD of ≤1% VAF with only 10 ng of input DNA, but only for exon 19 and 21 mutations [65]. Noteworthily, the T790M mutation has been reported to have a significantly higher LOD (8% VAF with 50 ng input and 2% VAF with 400 ng input), suggesting that it can be missed in some cases, especially when subclonal [7]. This has been reported on histological samples as well [69,70].

### 3.2. Performance on Different Cytological Samples

Because the bioptic material is often scant, it is convenient to be able to use whatever is available, or even to repurpose samples that would otherwise have been wasted (e.g., needle rinse, supernatant) [71,72,73]. The Idylla EGFR test has been performed, in different studies, on the whole range of cytological specimens: cell-block sections (unstained or stained with H&E), scraped smears (stained with Papanicolaou, Diff-Quik, H&E, or even immunocytochemistry), suspensions of the freshly aspirated material (fixed in CytoLit or unfixed, in PBS), and finally extracted DNA from sources as diverse as cell-block sections, cell pellets, pre-capture NGS libraries and stained smears (Table 1).

One important note is in order: while all other specimens appear interchangeable in terms of diagnostic yield, stained smears have been linked to false negatives and, less frequently, false positives [7,65,66]. De Luca and colleagues [66] found 2 false-negative L858R mutations on stained slides and noted the association with a high background fluorescence in the second channel of the Idylla EGFR cartridge, where the detection of the L858R mutation takes place. This background fluoresce was higher when the smears were stained with H&E and Papanicolaou and lower with Diff-Quik, and de-staining helped but did not eliminate it completely [66]. Arcila and colleagues found both false negatives and false positives [7]. These cases can usually be recognized at the manual review of the amplification curves, but orthogonal testing is required for confirmation.

Despite these problems with stained smears, sometimes the material is so scant that a smear is all that is available to determine the molecular phenotype of an NSCLC. In these contexts, pathologists are often reluctant to sacrifice their best (or only) diagnostic smear to molecular methods [66,74,75]. Because it is the smear with the highest tumor cellularity, however, the best smear is often the most suitable for molecular tests [76,77]. This problem may be solved by the digitalization of the smear, to capture its morphology indefinitely (for medico-legal or teaching purposes) [78]. The biological material will then be available for molecular tests.

Similarly, the only smear suitable for molecular tests may be stained with immunocytochemistry (most often with hematoxylin and diaminobenzidine) [79,80,81]. Among all the cases included in the present review, only one diaminobenzidine-stained slide was tested as input for the Idylla EGFR system, and it resulted in a false negative result [66]. However, a single case is not enough to draw conclusions and further studies on the matter are encouraged. Furthermore, when the alternative is between performing an EGFR test on suboptimal material and not performing it at all, a (false) negative result would not fundamentally alter the clinical management of the patient. A true positive, on the other hand, would give the patient access to a more effective treatment [82]. If supported by future studies, immunocytochemically-stained smears could also be digitized to allow sacrification of the biologic material for molecular tests [83].

### 3.3. Pitfalls of Idylla on Cytological Specimens

The Idylla EGFR cartridge assesses only 51 different exons 18–21 EGFR mutations [7]. All other mutations are not recognized by the system, so they will be false negatives by design [84]. Notably, some resistance mutations such as C797S and G724S are not detected by Idylla. Coupled with the relatively lower limit of detection for the T790M mutation, this makes Idylla less useful in the setting of EGFR-TKI resistance [7].

It should be pointed out that with cytological samples, as with histological samples, Idylla is sensitive to both a scarcity and an excess of input DNA. In the case of scarce DNA (high total EGFR Cq), Idylla can sometimes call a negative rather than an inadequate. Manual review of the curves is fundamental to identify these cases [7]. In the case of excessive input DNA (low total EGFR Cq), as is the case with cartridge overload or EGFR amplification, false positives may be called [7]. This is crucial to keep in mind when the input is not a canonical FFPE tissue slice or extracted DNA, but rather a scraped smear or a cell suspension, where precise quantitation of the input DNA and percentage of tumor cells can be difficult or impossible. In these cases, the automated Idylla callings should not be trusted and the amplification curves should always be manually checked [7].

Finally, as already discussed, care should be taken when using stained smears as input, for the associated risk of false negatives and false positives due to the background fluorescence introduced by the stain.

### 3.4. The Agility of Cytological Sampling

It is well known that it is best for the collection of the sample to be performed directly by the cytopathologist who will then evaluate it microscopically, and not by some other professional figure [85]. In the procedure known as rapid on-site evaluation (ROSE), the cytopathologist can smear the material on glass slides, air-dry them, stain them with a quick cytological stain (such as Diff-Quik) and evaluate them for adequacy on the spot, using a microscope [86,87,88,89]. While a morphological diagnosis can be rendered directly in some cases, the most important thing is that ROSE allows the cytopathologist to perform two crucial steps: sampling can be repeated on the spot if the first smears are inadequate or non-diagnostic, thus greatly reducing the rate of inadequate diagnoses, patient anxiety, and diagnostic delay; by integrating clinical, imaging (ultrasonography, computed tomography) and microscopic cytologic data, the material can be allocated in the best way possible [90,91,92]. For example, in suspect lymphoproliferative disorders, some material may be saved in phosphate-buffered saline (PBS) to perform flow cytometry [93], and in lymph nodal metastases of unknown primary, some material can be fixed in formalin to prepare a cell block for accurate immunohistochemical phenotyping [93,94,95,96]. This makes fine-needle aspiration more effective, reliable, and accurate [97,98,99].

Cytological sampling harvests fresh tissue and living cells [100,101]. This means that virtually any subsequent test can be performed on the material if it is stored correctly, from cytological (smears) to histological (cell blocks) preparations, with the whole array of ancillary tests at one’s disposal (immunocytochemistry, immunohistochemistry, flow cytometry, cytogenetics, molecular biology) [102,103,104,105,106,107]. One important factor that must not be overlooked in the context of molecular biology is that harvesting fresh (unfixed) material means that the quality of nucleic acids (both DNA and RNA) is at its best because no fixatives have been used and the risk of contamination is vastly reduced [108,109,110].

In the context of lung cancer, the most common contexts in which a cytopathologist is called to act are [72,111,112]: during bronchoscopy (with or without ultrasound guidance), where fine-needle aspirates and forceps biopsies can be performed; to perform a computed-tomography–guided fine-needle aspiration (CT-FNA); to perform fine-needle aspiration of a non-pulmonary mass, for example, a metastatic lymph node or suspect cutaneous metastasis; to analyze a fluid for malignancy in the context of exfoliative cytology (e.g., bronchoalveolar lavage, bronchial washing, pleural effusion).

In the first three cases, when a fine-needle aspirate is performed, then the cytologist has at its disposal the whole array of cytological techniques, ancillary techniques, and he can always fall back to histological techniques by preparing a cell block [113,114,115]. Performing rapid on-site evaluation complements and enhances the accuracy of the procedure.

However, even when the sampling is not cytological but histological (e.g., endobronchial forceps biopsy, core needle biopsy) the agile cytopathologist can employ quick cytological techniques for rapid on-site evaluation and specimen triage [116,117,118,119,120,121,122,123]. Small biopsies can be used to prepare the whole array of cytological specimens in several ways. For example, the biopsy can be used to prepare a touch imprint cytological glass slide, or it may be submerged in phosphate-buffered saline and delicately agitated to harvest some cells, before submerging it in the final container with fixative. Finally, after the bioptic sample has been fixed in formalin and collected to be processed histologically, the residual fixative may be centrifuged to harvest detached cells. The quality and quantity of DNA obtained with these methods is variable and has not been, to date, formally assessed in the literature.

Regarding fluids, regardless of how they are sent to the pathology lab (i.e., fresh or diluted with fixative), they are an important source of genetic material. Sometimes, they can be collected by the cytopathologist. In this regard, Al-Turkmani and colleagues [84] described their experience with using the Idylla KRAS test on pancreatic cyst fluid. In this case, an undetermined morphological diagnosis was greatly enhanced by the data provided by Idylla (KRAS mutation) using the aspirated cyst fluid.

### 3.5. Strengths and Weaknesses of the Idylla EGFR Test

The main advantage of the Idylla platform is its speed: less than two minutes of hands-on time and less than 2.5 h of total time to results. Because no batching is required (each sample is processed on its own), no additional time other than the running time of the cartridge is required. For comparison, in one of the studies included in this review, the average turn-around time was 2 days with Idylla versus 14–28 days with NGS [7]. If the freshly aspirated material is centrifuged and pipetted directly in the cartridge, the results can be ready in less than three hours from the biopsy [85]. Such speed is unparalleled by NGS and even by other PCR-based methods.

Additionally, Idylla is extremely easy to use if compared with NGS and PCR-based methods, and no specific training is required. This allows even peripheral laboratories without experienced molecular biologists to perform some molecular tests in-house rather than outsourcing them, dramatically decreasing turnaround time [66].

As De Luca and colleagues have shown, the cartridge can even be loaded directly by the cytopathologist with minimal training [85]. If the Idylla workstation is placed in the same room, or if the cartridge is run right away, then this means that the test results will be ready in a few hours from the sampling. This saves enormous amounts of time and money, because it is not only conceptually simpler, but it obviates the need for numerous intermediate time-consuming and costly steps. Because there is no need to prepare a cell block, at least one day of waiting time, several minutes of lab technician time, and all the reagents are saved. Then, there is no need to process the cell block, embed it in paraffin, cut the resulting block and stain it. Similarly, no biologist time or reagents are required because no extraction step is necessary. In some labs, due to batching or due to the need to ship formalin-fixed paraffin-embedded blocks or unstained sections to a more central laboratory, this procedure might take several days [85]. On the other hand, when the cytopathologist can load the cartridge and run the test on the spot, then the patient can have a combined morphological and molecular diagnosis on the same day of the aspirate. In addition to hastening clinical management and preventing therapeutic delays, this will also relieve the anxiety of the patient by shortening his waiting time [124].

Such an astonishing speed must come with compromises [125]. In fact, a single cartridge is more expensive than the same assay performed with other means, and, in comparison with NGS, the total number of mutations detectable is limited to the 51 included by design. Furthermore, only the EGFR gene is assessed, while the number of known driver mutations in NSCLC is steadily rising [6,39]. Other Idylla cartridges that might be useful in NSCLC exist (i.e., the KRAS and BRAF tests) [126], and another interesting cartridge is in development (currently for research use only), targeting ALK, ROS1, RET, and NTRK1/2/3 rearrangements as well as MET exon 14 skipping. However, each cartridge is a closed system that runs independently from the other ones, with a different input sample and the costs are additive. What is a strength for the single case (i.e., the possibility to rinse the needle directly in the cartridge and have the results in a few hours) becomes a weakness when the cases cannot be batched, the material cannot be used for multiple tests, and the costs are not amortized.

As with everything, and—for the case in point—molecular tests, perfection does not exist and some choices have to be made. Each pathology service has different needs, and the optimal instruments have to be chosen on a per-case basis.

## 4. Materials and Methods

Two authors (Alessandro Caputo and Angela D’Ardia) independently searched 4 databases (PubMed, EMBASE, Scopus, Google Scholar) from inception to 15 February 2021 using the following queries:

- PubMed: idylla (lung OR NSCLC OR pulmonary) (cytology OR cytological OR smear OR FNAB OR FNAC OR needle OR aspiration)
- EMBASE: idylla:ti,ab,kw AND (lung:ti,ab,kw OR nsclc:ti,ab,kw OR pulmonary:ti,ab,kw) AND (cytology:ti,ab,kw OR cytological:ti,ab,kw OR smear$:ti,ab,kw OR fnab:ti,ab,kw OR fnac:ti,ab,kw OR needle:ti,ab,kw OR aspiration:ti,ab,kw)
- Scopus: idylla AND (lung OR NSCLC OR pulmonary) AND (cytology OR cytological OR smear OR FNAB OR FNAC OR needle OR aspiration)
- Google Scholar: idylla lung cytology OR cytological OR smear OR FNAB OR FNAC OR needle OR aspiration

Studies were screened first based on the title, then on the abstract, and finally on the full-text, where available. The present review included original studies using cytological specimens of NSCLC as input to the Idylla™ EGFR assay (Biocartis, Mechelen, Belgium). Review articles were excluded but their reference lists were hand-searched for potentially relevant articles to include in this review. The reference lists of all included articles were also recursively hand-searched.

Studies were appraised and data were extracted independently by two authors (AC and AD’Ar). When needed, authors of the included studies were contacted via email. Disagreements were resolved by discussion among the two authors responsible for the search, and, when needed, with a third author (PZ).

## 5. Conclusions

Usage of the Idylla EGFR test as a first (triage) step seems reasonable, given its high specificity and the relatively high prevalence of EGFR mutations (15–40%) in non-squamous NSCLC [6,7]. If no mutations are identified by Idylla, further NGS testing is not compromised; however, for EGFR-mutated patients, a positive Idylla result will mean that therapy can be started in a matter of days rather than weeks [7]. Thus, a workflow where actionable and common mutations are tested first and fast, leaving rarer mutations for subsequent comprehensive profiling, seems the Cmost effective (albeit more expensive) approach to NSCLC phenotyping.

## Figures and Tables

**Table 1:** Input material used for the Idylla EGFR assay in the studies included in the present review. FFPE: formalin-fixed and paraffin-embedded. H&E: hematoxylin and eosin. ICC: immunocytochemistry. PBS: phosphate-buffered saline. NGS: next-generation sequencing.

Input Material	No. of Cases	References
Cell-block (FFPE) section	181	[7]
Scraped stained smear		
Papanicolaou	11	[65,66]
Diff-quik	36	[7,65,66]
H&E	21	[66]
ICC	1	[66]
Suspension		
In CytoLit	62	[7]
In PBS	28	D’Ardia 2021 (unpublished)
Extracted DNA		
from cell-block	25	[7,66]
from pellet	14	[7]
pre-capture NGS library	16	[7]
from stained smear	76	[65]

## Data Availability

Not applicable.
